# Optical Characterization of Biological Tissues Based on Fluorescence, Absorption, and Scattering Properties

**DOI:** 10.3390/diagnostics12112846

**Published:** 2022-11-17

**Authors:** Omnia Hamdy, Zienab Abdel-Salam, Mohamed Abdel-Harith

**Affiliations:** 1 Engineering Applications of Lasers Department, National Institute of Laser Enhanced Sciences, Cairo University, Giza 12613, Egypt; 2 Laser Applications in Metrology, Photochemistry and Agriculture Department, National Institute of Laser Enhanced Sciences, Cairo University, Giza 12613, Egypt

**Keywords:** laser, biological tissue monitoring, fluorescence, absorption, scattering

## Abstract

Optical diagnostics methods are significantly appealing in biological applications since they are non-destructive, safe, and minimally invasive. Laser-induced fluorescence is a promising optical spectrochemical analytical technique widely employed for tissue classification through molecular analysis of the studied samples after excitation with appropriate short-wavelength laser light. On the other hand, diffuse optics techniques are used for tissue monitoring and differentiation based on their absorption and scattering characteristics in the red to the near-infrared spectra. Therefore, it is strongly foreseen to obtain promising results by combining these techniques. In the present work, tissues under different conditions (hydrated/dry skin and native/boiled adipose fat) were distinguished according to their fluorescence emission, absorption, and scattering properties. The selected tissues’ optical absorption and scattering parameters were determined via Kubelka–Munk mathematical model according to the experimental tissue reflectance and transmittance measurements. Such measurements were obtained using an optical configuration of integrating sphere and spectrometer at different laser wavelengths (808, 830, and 980 nm). Moreover, the diffusion equation was solved for the fluence rate at the sample surface using the finite element method. Furthermore, the accuracy of the obtained spectroscopic measurements was evaluated using partial least squares regression statistical analysis with 0.87 and 0.89 R-squared values for skin and adipose fat, respectively.

## 1. Introduction

The use of optical spectroscopic techniques has grown significantly in various biological investigations. These techniques are reliable, functional, safe, and closely related to physiological alterations in biological tissue [1,2]. Additionally, research into how light travels through tissues provides information on tissue architecture and physiology that can directly quantify tissue damage or abnormalities [3]. Due to its potential for non-destructive medical diagnostics and treatment, light interaction with biological tissues is receiving more and more interest. Laser-induced fluorescence (LIF) is an optical spectroscopic approach with high precision, simplicity, and less destructive consequences. Laser-induced fluorescence (LIF) is a spectrochemical analytical technique extensively used to classify tissue components such as proteins and other metabolic mediators by interpreting the examined tissue’s light emission [4]. It has been successfully employed in detecting fraud in different food products such as meat [5], fish [6], and olive oil [7]. Based on the differences in optical characteristics between normal tissue and carious lesions, LIF spectroscopy has been employed for the early diagnosis of human carious lesions. The optical system included a visible-band light source, a hyperspectral camera, and a specially developed digital image processing algorithm [8].

Moreover, the application of LIF in vital medical and biological fields, including malignancy detection, is of great interest to the scientific community [9]. Additionally, LIF imaging is considered a precise laser technology with high spatial and temporal resolution molecular visualization characteristics. Therefore, it has been broadly utilized in fluid mechanical processes, sprays, and combustion systems to monitor species concentration, mixture fraction, fluid flow visualization, and temperature via a common technique called planar laser-induced fluorescence (PLIF) [10]. Additionally, fluorescence-based tumor imaging has the potential for detecting cancer in situ. For example, LIF imaging made it possible to distinguish between malignant and healthy head and neck tissues by observing the changes in autofluorescence properties [11].

Biological tissue’s optical characteristics, primarily described by tissue absorption and scattering coefficient factors, affect how light interacts with and propagates through it [12]. Light scattering provides details about the micrometric-size objects (for example, molecular weight) that contribute to light scattering in the sample. On the other hand, the absorption of light in a tissue sample provides quantitative identification of the molecules present in that sample, their concentration, and their local environment [13]. Due to its high tissue penetration, near-infrared light is chosen for working with biological tissues; photons could penetrate deeply into tissue in the red and near-infrared NIR spectral window (between 600 and 900 nm) because water and hemoglobin absorb light at comparatively low levels. As a result, when tissue is illuminated with NIR light, the transmitted and/or reflected signal can be logically described as a diffusive process. Therefore, the essential information used in estimating the optical characteristics of the tissue, and thus in tissue monitoring and characterization operations, is diffuse reflectance and transmittance values [14].

Various experimental techniques can be utilized to measure tissue-diffused light. Integrating spheres are the most popular tools for estimating biological samples’ total transmittance and diffuse reflectance [15]. While acquiring the tissue’s diffusion data, other methods employ various configurations of light sources and detectors [16,17]. The gathered observations are then used to estimate optical properties in mathematical models. Based on the radiative transport equation (RTE) of light propagation in biological tissue, various analytical and computational techniques can predict tissue’s optical properties from dispersed light readings. When used to describe how photons travel within tissues over space and time, RTE is thought of as an approximation to the standard Maxwell’s equations. Several assumptions, such as diffusion approximation, have been applied to make it simpler to reach an analytical and/or numerical solution to RTE [18]. Accordingly, various analytical models can be utilized. These models can be forward models, such as the Monte Carlo method [19] or inverse models, including Kubelka–Munk (KM) [20], inverse Monte Carlo (MC) [21] and inverse adding doubling (IAD) methods [22]. However, KM is the most extensively applied transport model owing to the fact that the scattering and absorption coefficients can be directly depicted in terms of the measured reflection, transmission, and sample thickness, which is by far its most significant benefit over more complex models. Therefore, its parameters are frequently employed in the discipline of medical physics [23]. Moreover, it has been used to investigate various materials, such as paints, colorful polymers, protective coatings, and biological tissues [24].

Understanding the ideal use of lasers in numerous diagnostic and therapeutic applications, including photodynamic therapy, optogenetics, and biosensing, depends on assessing tissues’ optical characteristics [25,26,27,28]. It also plays a crucial role in identifying tissues [29]. As a result, numerous investigations have been suggested in that field of study. For example, Firbank et al. [30] investigated the optical parameters of pig skulls in the 650–950 nm spectral window. Moreover, the optical properties of mice skull bone (fresh native) in the 455 to 705 nm range were determined by [31]. An ex vivo experiment to obtain the optical properties of normal and thermally coagulated chicken liver at several laser wavelengths was implemented by Hafeez-Ullah et al. [20]. Optical properties of rabbit brains in the red and near-infrared spectral range were also evaluated in vivo under various settings (postmortem, frozen, and formalin-fixed conditions) to investigate the impact of prolonged storage on the tissue’s optical characteristics [32]. Optical coefficients and the refractive index of rabbit head tissues were also ex vivo estimated at selected laser wavelengths using the KM model and IAD method [33]. Recently, an analytical comparison between the performance of single- and double-integrating sphere configurations on optical parameters prediction was proposed using Monte Carlo modeling and a synthetic tissue phantom [19].

The primary goal of the present work is the noninvasive classification and characterization of biological tissues based on their optical and spectrochemical characteristics. Therefore, tissues’ fluorescence, absorption, and scattering properties were evaluated under different conditions: skin (hydrated and dry) and adipose fat tissue (native and boiled). In addition to the variations in the optical absorption and scattering coefficients of the studied samples, the LIF data was successfully employed to differentiate between hydrated/dry skin and native/boiled adipose tissue by comparing the emission intensity under different tissue conditions. Such intensity is highly affected by the possible scattering events within the tissue. Accordingly, combining the two methodologies empowers more representative tissue differentiation and hence a precise optical diagnosis approach.

## 2. Materials and Methods

### 2.1. Tissue Selection, Collection, and Preparation

Separate samples of bovine adipose tissue and chicken breast skin were purchased from various butcher shops near the campus of Cairo University. Samples were examined within an hour after the animals were slaughtered. Of the 50 samples, 25 chicken breast skin and 25 adipose fat tissue samples were investigated. Skin samples were 2 ± 0.5 mm thick, while adipose fat samples were 3 ± 0.4 mm thick, as measured using a digital micrometer (Digimatic micrometer, Mitutoyo Corporation, Sakado, Japan). The sample’s thickness has a significant effluence on the experimental result. However, the optical inhomogeneities in the sample responsible for the scattering must be uniformly distributed throughout and much smaller than the sample’s thickness. Therefore, the sample thickness was selected to enable appropriate transmission and reflection measurements required for applying the KM method [34].

The samples were cleaned with running water and dried with paper towels prior to the experimental experiments. The same skin sample was left to dry for 24 h to have a dry skin sample. Hence the optical parameters could be affected, reflecting the new status of the skin sample. Boiled adipose fat samples were created by boiling the native fat samples in distilled water for one minute. The current study does not directly include any contact with living animals. Therefore, no formal ethical approval was needed. The studied biological tissue samples were collected from animals that had already been slaughtered for the purpose of producing commercial food.

### 2.2. Laser-Induced Fluorescence Spectroscopy (LIF)

In a typical LIF practice, fluorophore molecules in the examined samples absorb the input laser photons at a specific wavelength. As a result, fluorescence emission takes place at longer wavelengths due to the Stokes shift resulting from an energy loss in a non-radiative decay [35]. An electron is promoted to a higher energy level equivalent to the absorbed photon’s energy when a molecule is in its ground state and absorbs a photon with enough energy. If the electron moves through a relaxation pathway and emits a photon while returning to its starting condition, it is referred to as photoluminescence. The two types of photoluminescence are fluorescence and phosphorescence. Fluorescence occurs when an electron from a singlet excited level radiatively decays to the ground singlet state within a typical decay period of 10^−10^ s to 10^−7^ s. In phosphorescence, on the other hand, the electron molecules return to their ground state through a triplet state and take a much longer time [36].

**Figure 1:**
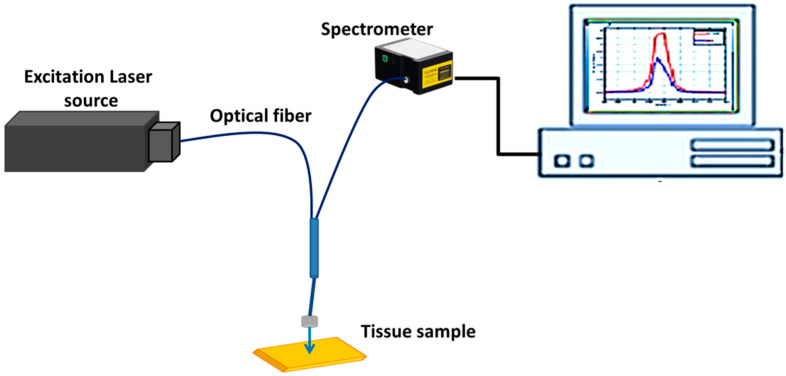
Schematic for the laser-induced fluorescence experimental system.

In the current study, the laser used in the LIF setup is a CW (DPSS) laser (Changchun new industries optoelectronics Tech Co., Ltd. Changchun, Jilin, China) delivering laser light of wavelength 266 nm (Δλ = 2.5 nm) and 5 mW power. A specific annular Y-type optical fiber provides the laser beam perpendicular to the sample through one branch. At the same time, the released fluorescent light is received in the other that feeds it to the entrance slit of the miniature spectrometer (USB2000 FLG, Ocean Optics, Largo, FL, USA). The Spectra Suit software (Ocean Optics, USA) was used to control the spectroscopic system and display and record the acquired spectra on the PC. Further data and relevant spectra were processed via commercial software (Origin, Origin Lab. Corp., Northampton, MA, USA, version 9). Schematic of the experimental setup is presented in Figure 1.

Furthermore, the LIF spectra of the examined tissue samples were validated in the current study using the multivariate calibration technique partial least squares regression (PLSR). PLSR is used to model the response variable of highly correlated datasets with a large number of predictor variables by generating new predictors known as “components” [37]. Unlike the principle component regression (PCR) method, the response variable was considered when building the PLSR components, which show the observed variability in the predictors [38,39]. PLSR of the acquired LIF spectra was implemented based on a Matlab function called “plsregress”. Partial least squares regression of the response matrix on the predictor variables matrixes was conducted after determining the PLS component count. The predictor and response loadings were then given, and the R-squared (R^2^) value was estimated. Regression is generally used to find the best-fit line to a dataset. As a result, ensuring that the measured data is correlated and that there are no anomalies [40]. Consequently, the PLSR method was implemented to evaluate the correlation of the experimental LIF data and ensure the absence of any odd measurements.

### 2.3. Tissue Reflectance and Transmittance Measurements via Integrating Spheres

**Figure 2:**
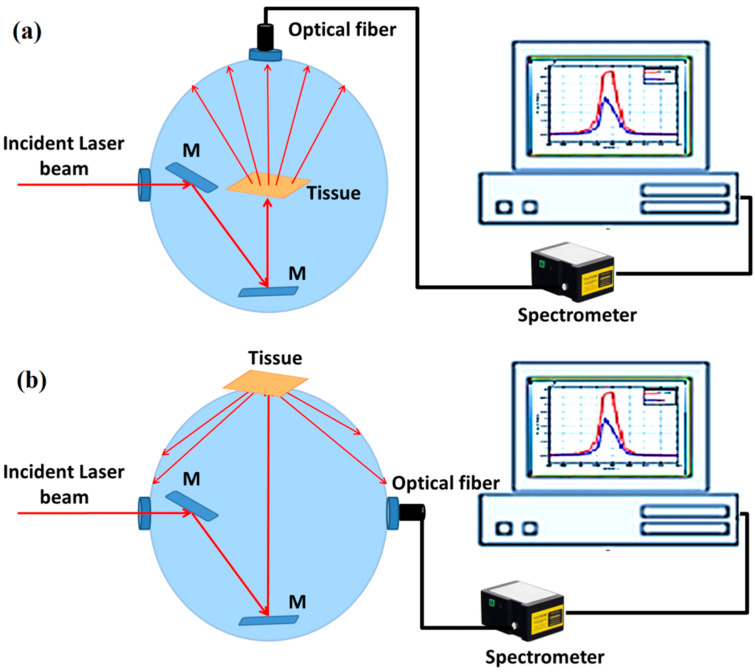
Single integrating sphere to measure (**a**) total transmittance and (**b**) diffuse reflectance.

The diffuse reflectance and transmittance of the examined tissues are measured via an integrating sphere (McPherson, KS, USA). The integrating sphere is a hollow spherical cavity coated with highly diffusive reflective material to ensure that all light entering it is reflected. It has two sample locations, one for measuring reflectance and the other for measuring transmittance, as shown in Figure 2. Therefore, the same experiment must be run twice when using a single integrating sphere; the first implementation is for measuring tissue reflectance, and the second is for measuring transmittance dependent on the positions of the detector and sample, as clarified in the figure.

The output signal is detected using a measuring detector (Toshiba TCD1304AP with Sony ILX511 2048 Linear CCD array) linked to a digital fiber spectrometer (STDFSM, Touptek Photonics Co., Ltd., Zhejiang, China) which is connected to a computer for the further processing. Data analysis was performed using Matlab R2018a (MathWorks, Inc., Natick, MA, USA). Different laser wavelengths (808, 830, and 980 nm) were utilized as the incident laser beam provided by 100 mW power infrared semiconductor laser modules (LW-808-100-C12, LW-830-100-C12, and LW-980-100-C12, LAMBDAWAVE Technika laserowa, Wroclaw, Poland).

### 2.4. Estimating Tissue’s Optical Parameters

Tissue diffuse measurements are used in a mathematical model to estimate optical properties. The KM model anticipates two interior fluxes passing through the tissue, one moving in the same direction as the incident radiation and the other moving in the opposite way. Two coefficients are suggested to illustrate the absorption and scattering of diffuse radiation, respectively (A_KM_ and S_KM_). The following equations connect these coefficients to tissue transmittance and reflectance;

(1) $$R_{d}=\frac{\sinh(S_{\mathrm{KM}}\;y\;D)}{x\;\cosh\left(S_{\mathrm{KM}}y\;D\right)+y\;\sinh(S_{\mathrm{KM}}\;y\;D)\;}$$

(2) $$T_{d}=\frac{y}{x\;\cosh\left(S_{\mathrm{KM}}y\;D\right)+y\;\sinh(S_{\mathrm{KM}}\;y\;D)\;}$$

where D is the slab’s optical thickness. The parameters x and y can be written as:

(3) $$\;x=\frac{1+R_{d}^{2}-T_{d}^{2}}{2R_{d}}$$

(4) $$y=\sqrt{x^{2}-1\;}$$

The following equation is then used to relate S_KM_ and A_KM_ with the sample’s absorption and scattering coefficients:

(5) $${\;A}_{\mathrm{KM}}=2\mu_{a}$$

(6) $${\;S}_{\mathrm{KM}}=\frac{3}{4}\mu_{s}\left(1-g\right)-\frac{1}{4}\mu_{a}$$

where ${\overset{`}{\mu }}_{s}=\left(1-g\right)\mu_{s}$ is the reduced scattering coefficient and g is the anisotropy factor [41].

### 2.5. Fluence Rate Modeling and Simulation Using COMSOL Multiphysics Software

Using the diffusion approximation of the RTE, the diffusion equation is solved using finite elements to calculate the distribution of optical fluence on the tissue surface.

(7) $$\frac{\partial Ф\left(\vec{r},\;t\right)}{c\partial t}+\mu_{a}Ф\left(\;\vec{r},\;t\right)-\nabla .\left[D\nabla Ф\left(\vec{r},\;t\right)\right]=S\left(\vec{r},\;t\right)$$

where $D=\frac{1}{3\left(\mu_{a}+{\overset{`}{\mu }}_{s}\right)}$ is the tissue diffusion coefficient, $S\left(\vec{r},\;t\right)$ represents the source term, and $Ф\left(\vec{r},\;t\right)$ is the fluence rate. However, the Helmholtz equation in the COMSOL Multiphysics program can display this diffusion equation in the steady-state, as follows:

(8) ∇(−c∇u)+au=f

Identifying the parameters with equation (11) yields:

(9) $$u=Ф,\;a=\mu_{a}\;c=D=\frac{1}{3\left(\mu_{a}+{\overset{`}{\mu }}_{s}\right)},\;S=f$$

**Figure 3:**
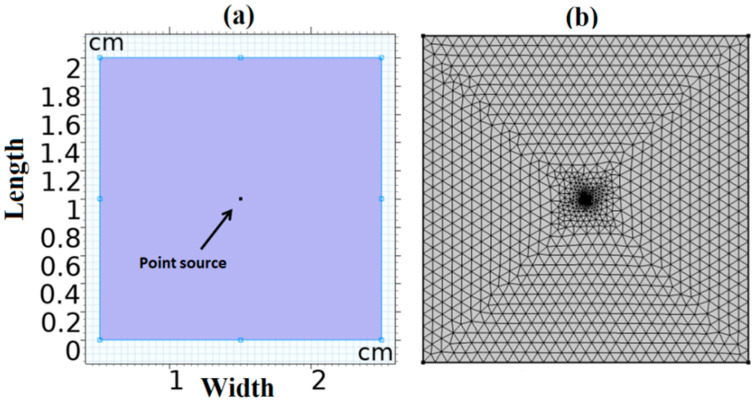
The reconstructed model for the examined tissue sample. (**a**) Model geometry and (**b**) finite element mesh.

Under the COMSOL Multiphysics software environment, the finite element method is used to solve Equation (8) for the optical fluence rate. The optical parameters affect how fluence is distributed along tissue borders. Therefore, offering graphic projections of these distributions can provide biological diagnostics with a visual component. Figure 1 illustrates the geometry and the defined finite element mesh of the tissue model constructed in the COMSOL program. The incident laser beam is obtained by a point source at the model’s center, as illustrated in Figure 3a. Consequently, for solving the diffusion equation (Helmholtz equation in COMSOL module), the defined mesh size becomes finer at the center point as shown in Figure 3b.

For each simulation execution, the calculated absorption coefficient and reduced scattering coefficient (denoted by “mua” and “musp,” respectively, in the COMSOL tutorial, presented as Supplementary Materials Video S1) have to be entered as the global definition in the model. In addition, another necessary parameter, “A”, the internal reflectance factor, is entered. This factor depends on the effective reflection coefficient R_eff_ as A = (1 + R_eff_)/(1 − R_eff_)) and is related to the boundary absorption/impedance term q, where q = 1/2A. Finally, the index mismatch between the diffusing medium and air is calculated using R_eff_ and is provided by [42]:

R_eff_ = −1.44n^−2^ + 0.7 n^−1^ + 0.063n + 0.668 (10)

where n = n_in_/n_out_ is the inside-to-outside diffusing medium’s refraction index ratio, accordingly, and q between every examined tissue and air was calculated. (Supplementary Materials Video S1 shows a video example for one execution of the optical field dynamics when impeding tissues with different optical properties).

## 3. Results

The current study identified tissues under various conditions based on their fluorescence emission, absorption, and scattering characteristics.

### 3.1. Laser-Induced Fluorescence Characteristics

**Figure 4:**
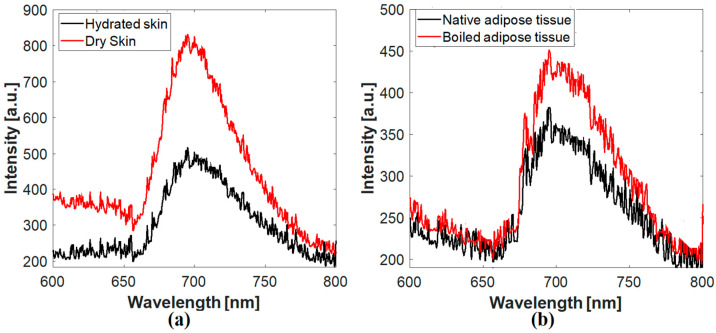
The fluorescence emission spectra of the examined samples. (**a**) Skin and (**b**) adipose tissue.

The obtained LIF spectra of the investigated tissues are presented in Figure 4. The main fluorescence peak for the two tissue types (i.e., skin and adipose fat) was recorded at 700 nm, representing the porphyrin content in that tissue [43]. It is evident that changing the tissue condition (drying in the case of skin and boiling in adipose fat) causes an increase in fluorescence intensity. To avoid sample surface inhomogeneity, the examined fluorescence, reflectance, and transmittance measurement is the average of 10 spectra obtained from 10 locations on each sample’s surface. Although the whole-body imaging of fluorescent cells on virtually all organs is possible using fluorescent protein-based imaging techniques [44], the present LIF method depends on the intrinsic fluorescence emission of the tissue (stimulated at 266 nm and emitted at around 700 nm).

#### Statistical Validation via PLSR

**Figure 5:**
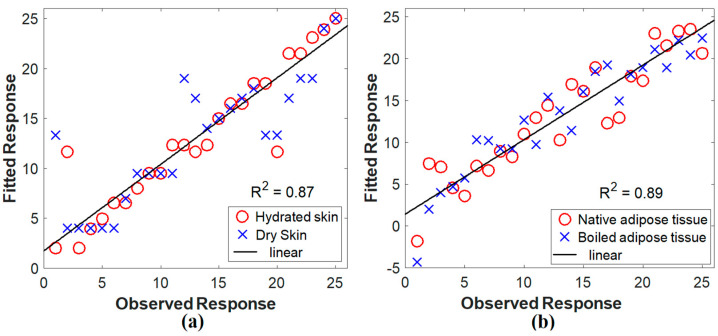
PLSR of the LIF measurements. (**a**) Skin and (**b**) adipose fat.

When there are various highly correlated and/or collinear predictor variables, PLSR is used to model the response variable. The technique creates new predictor variables, referred to as components, by linearly combining the initial predictor variables. It develops components to describe observed variability in predictor variables while accounting for the response variable. Through PLSR, the experimental measurements’ correlation was assessed. It is used to find the line that fits the dataset the best and ensure that the measured data is correlated and that no unusual values exist. The relationship between the actual and predicted values according to our measured spectroscopic data is shown in Figure 5. The model’s performance was good, as indicated by the calculated R^2^-values of 0.87 and 0.89 for skin and adipose fat tissue samples, respectively.

### 3.2. Optical Absorption and Scattering Properties

Measurements of tissue diffuse reflectance and transmittance made experimentally were used to compute the tested samples’ optical coefficients at the selected laser wavelengths. Based on KM model calculations, the studied tissues’ optical absorption and reduced scattering coefficients were acquired. The average values with their standard deviation are shown in Table 1. All measurements were repeated five times.

Compared with the literature, our calculated optical absorption and scattering coefficients of adipose fat and skin samples agree with those of Bashkatov et al. [45,47] and Beek et al. [46] at the same or near wavelengths. The slight difference could result from the samples’ source (using samples from other animals, pigs, or rats), storage and preparation methods, and the employed mathematical models and experimental arrangements.

#### Fluence Rate Distribution

**Figure 6:**
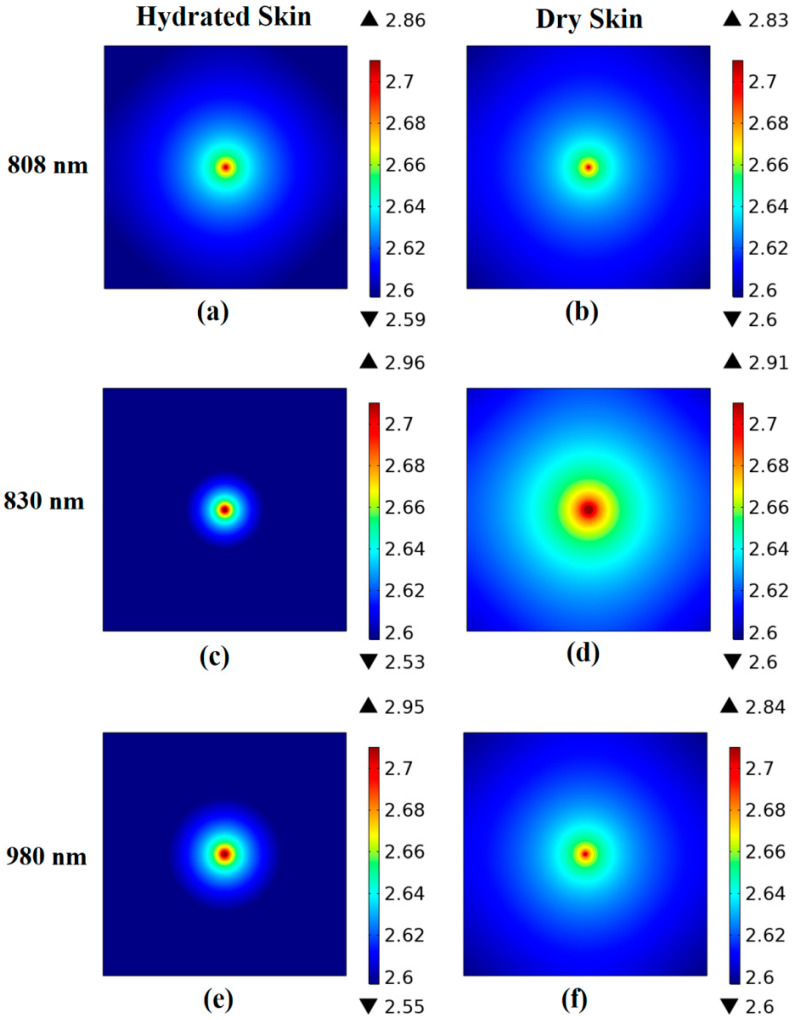
The fluence rate distribution along skin samples surface at each utilized wavelength. (**a**) Hydrated skin at 808 nm, (**b**) dry skin at 808 nm, (**c**) hydrated skin at 830 nm, (**d**) dry skin at 830 nm, (**e**) hydrated skin at 980 nm, (**f**) dry skin at 980 nm.

**Figure 7:**
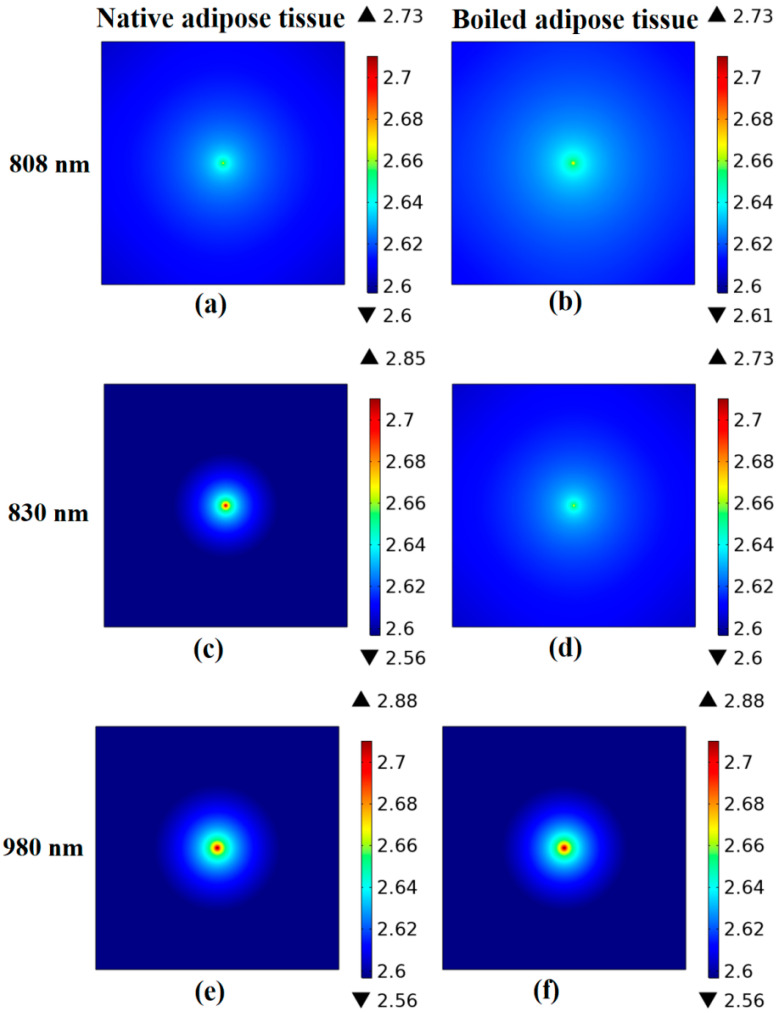
The fluence rate distribution along adipose tissue samples surface at each utilized wavelength. (**a**) Native adipose tissue at 808 nm, (**b**) boiled adipose tissue at 808 nm, (**c**) native adipose tissue at 830 nm, (**d**) boiled adipose tissue at 830 nm, (**e**) native adipose tissue at 980 nm, (**f**) boiled adipose tissue at 980 nm.

The fluence rate at the sample’s outer layer was determined using the finite element method. The results of skin and adipose fat tissues are displayed in Figure 6 and Figure 7, respectively. The minimum and maximum logarithmic values of the resultant fluence rate log(Ф) are shown within the color bar of the figures. These values differ with the tissue condition and the utilized wavelength. For example, the distribution of the optical fluence rate appears wider and more diffusive in the dry skin and boiled adipose fat samples. On the other hand, it is focused and collimated in the native tissues.

## 4. Discussion

The complexity of biological systems has made it possible to develop and validate a number of label-free methodologies, especially using natural fluorophores. Fluorescence spectrometry is now a common technique in many biomedical fields, such as biosensing, bioimaging, and drug discovery. It offers a flexible replacement for traditional studies that require radioactive labels. The LIF method was first used to assess the status of biological tissues by monitoring endogenous fluorophores in the 1990s. It has been employed to detect cancer in its early stages. It is also applied in atherosclerosis, renal and urolithiasis disorders, skin diseases, and early stages of tooth decay and to detect fungus in addition to its use in gynecological disorders and neoplasms [48]. This work’s main objective is the noninvasive classification and characterization of biological tissues according to their optical and spectrochemical characteristics. As a result, the tissues’ fluorescence, absorption, and scattering properties were evaluated under various conditions.

It is evident that many factors, including dynamic quenching, resonance energy transfer, and sequential scattering events, significantly influence the intensity and spectral characteristics of the released fluorescence in scattering media, such as biological tissues [49]. For example, as shown in Figure 4, changing tissue conditions by drying (in the skin) and boiling (in adipose fat) caused an increase in the intensity of the obtained fluorescence emissions. Such an increase may be due to varying the water content in the sample after the applied modification, which may alter the amplitude of the emission spectra and affect the absorption characteristics as well [50]. Additionally, the photostability of the fluorescent molecules, which induces irreversible degradation or “photobleaching,” is greatly affected by the fluorophore environment and the utilized excitation wavelength [51]. Moreover, the fluorescence from biological tissues incorporates information about scattering and absorption as well as intrinsic fluorescence, which is the fluorescence from an optically thin sample of pure fluorophores. Therefore, the interaction of scattering and absorption can substantially alter intrinsic spectral characteristics. These distortions can be addressed by combining a photon-migration-based image with data from fluorescence and reflectance spectra acquired concurrently. Consequently, disentangling the effects of scattering and absorption can minimize or eliminate the fluctuations of the emitted fluorescence [52].

Additionally, boiled adipose fat differs from native fat in terms of its macroscopic and microstructural features [53]. This occurs as it is released due to boiling from its connective tissue cells, namely “adipocytes”, which are specialized for fat storage, and its oxidation also increases [54]. Such characteristics may also explain the variations that were observed in the absorption and scattering properties of adipose fat after boiling. Laser light propagation in biological structures suffers from forward and multiple scattering due to the diversity in refractive indices and scattered particle sizes across tissue layers. These phenomena are closely related to the movement of light through biological material. Furthermore, the appearance, structure, and constituents of biological tissue, such as cell membranes and organelles, influence how light scatters within the tissue.

The optical coefficient values from the suggested results generally match those from relevant publications that used the same or near wavelengths [46,55,56,57]. At the same time, there could be some differences in the values owing to factors such as storage requirements and tissue preparation techniques [2,32]. Additionally, the drying of the tissue during the measuring processes can influence some of the calculated values [58]. Moreover, for every wavelength, the variation in optical parameter values provided to the diffusion equation induced the difference in fluence rate distribution at the tissue surface. Due to decreased scattering and higher penetration, the fluence rate at the tissue surface became less dispersed for longer wavelengths. Therefore, the highest fluence rate is attained at 980 nm because of the reduced scattering and improved transmission (see Figure 6 and Figure 7). However, this behavior is changed by changing tissue conditions facilitating the differentiation process. Finally, the PLSR method was utilized to validate the accuracy of the obtained fluorescence spectra showing acceptable R^2^ values in the two studied tissues. It is important to note that PLSR was not used to reflect the suggested method’s differentiation performance. However, it was used to determine the correlation between the multiple spectral observations that were acquired.

## 5. Conclusions

Tissue contents, such as hemoglobin/melanin concentration, oxygen saturation, lipid concentration, and cell nucleus size, which are crucial indications of tissue health, are closely related to the tissue’s fluorescence, absorption, and scattering properties. Additionally, the optical parameters affect how the optical fluence rate is distributed throughout the tissue. Therefore, providing information about these distributions is crucial for making an appropriate diagnosis. Accordingly, the present study aimed to non-invasively classify and characterize biological tissues according to their optical and spectrochemical properties. Under various conditions, skin and adipose fat tissue fluorescence, absorption, and scattering characteristics were evaluated.

Moreover, the fluence rate distribution at the tissue surface was modeled based on the finite element solution of the light diffusion equation providing a criterion for tissue differentiation. The obtained fluence rate distribution at the boundary of each tissue sample clearly demonstrates the difference in optical characteristics (absorption and scattering) between samples of normal/dry skin and native/boiled adipose fat. Additionally, the tissue fluorescence emission related to porphyrin content (i.e., the fluorescence peak at 700 nm) decreased after changing the tissue condition by drying and boiling. Consequently, the sample condition has a significant impact on the optical characteristics. The results demonstrate the importance of optical parameter values and fluence rate distribution at the tissue surface in differentiating tissues. As a result, they can help in the diagnostic process by being used in image reconstruction processes in some medical imaging techniques, such as diffuse optical imaging and biophotonics applications.

## Figures and Tables

**Table 1:** The calculated optical coefficients of the studied samples at different wavelengths.

	Tissue	Optical Parameters					
		µ_a_ [cm^−1^]			$\mu_{s}^{\prime }$ [cm^−1^]		
		808 nm	830 nm	980 nm	808 nm	830 nm	980 nm
This work	Hydrated skin	0.84 ± 0.47	2.68 ± 0.03	2.01 ± 0.04	26.2 ± 1.59	29.8 ± 0.02	32.5 ± 0.05
	Dry skin	0.5 ± 0.01	0.14 ± 0.01	0.48 ± 0.02	24.1 ± 0.04	38.7 ± 0.04	26.1 ± 0.07
	Native adipose tissue	0.34 ± 0.14	1.7 ± 0.01	1.61 ± 0.017	11.2 ± 018	19 ± 0.01	24 ± 0.02
	Boiled adipose tissue	0.13 ± 0.04	0.34 ± 0.15	1.63 ± 0.018	13.2 ± 0.06	11.1 ± 0.16	24.2 ± 0.03
Bashkatov et al. [45]	Adipose tissue	0.8 ± 0.2	1 ± 0.4	1.2 ± 0.5	11 ± 3	12 ± 4	13 ± 4
Beek et al. [46]	Rabbit skin	0.7 ± 0.07 (at 790 nm)			18.4 ± 0.05 (at 790 nm)		
	Piglet skin	1.6 ± 0.1 (at 850 nm)			14.3 ± 1.5 (at 850 nm)		

## Data Availability

Not applicable.

## Supplementary Materials

The following supporting information can be downloaded at: https://www.mdpi.com/article/10.3390/diagnostics12112846/s1, Video S1: A Video example for one execution of the optical field dynamics when impeding tissues with different optical properties.
